# Severe Myasthenic Manifestation of Leptospirosis Associated with New Sequence Type of *Leptospira interrogans*

**DOI:** 10.3201/eid2505.181591

**Published:** 2019-05

**Authors:** Matthias Tomschik, Inga Koneczny, Anna-Margarita Schötta, Sebastian Scharer, Merima Smajlhodzic, Paloma Fernandes Rosenegger, Martin Blüthner, Romana Höftberger, Fritz Zimprich, Gerold Stanek, Mateusz Markowicz

**Affiliations:** Medical University of Vienna, Vienna, Austria (M. Tomschik, I. Koneczny, A.-M. Schötta, S. Scharer, M. Smajlhodzic, P.F. Rosenegger, R. Höftberger, F. Zimprich, G. Stanek, M. Markowicz);; Medizinisches Versorgungszentrum Labor PD Dr. Volkmann und Kollegen GbR, Karlsruhe, Germany (M. Blüthner)

**Keywords:** myasthenia gravis, myasthenic crisis, leptospirosis, Leptospira interrogans, Asia, Austria, bacteria

## Abstract

We report the rapid development of a myasthenic crisis as the first-time manifestation of myasthenia gravis. The symptoms developed in the course of acute leptospirosis associated with a new sequence type of *Leptospira interrogans*. Antibiotic treatment led to rapid amelioration of myasthenia.

Leptospirosis is a global zoonotic disease, endemic in tropical areas, and caused by spirochetes of the genus *Leptospira* (*1*). Involvement of the nervous system is rare, and only single cases involving aseptic meningitis, encephalitis, movement disorders, neuritis, and polymyositis, among others (*2*,*3*), have been reported. We report a case of leptospirosis leading to a myasthenic crisis and subsequent diagnosis of a rare form of myasthenia gravis (MG) in a previously healthy traveler returning to Austria from Southeast Asia.

In August 2017, a previously healthy man, 32 years of age, visited an emergency department in Vienna, Austria, because of generalized weakness, malaise, double vision, and a 2-day history of fever and diarrhea. His medical history was unremarkable, and he was not taking any medication. Three days before the onset of symptoms, he had returned from a 4-week vacation in Vietnam and Thailand, where he swam in a waterfall pool and came into contact with indigenous animals. In particular, he helped to wash elephants in northern Thailand 1 week before his return. He reported feeling well upon arrival in Austria and even went white-water rafting in an alpine area the next day without experiencing weakness or malaise. However, 1 day later he noticed fatigability in his legs when walking up stairs, and nonbloody diarrhea developed. The diarrhea subsided, but other symptoms progressively worsened until he had trouble swallowing and walking.

A physical examination at the emergency department revealed elevated temperature (38.0°C), pulse (138 bpm), and blood pressure (146/104 mm Hg). The patient displayed typical myasthenic symptoms, including bilateral ptosis, Cogan’s lid twitch signs, bilateral weakness of ocular movements, and dysarthria. Symptoms worsened with prolonged talking, including dysphagia and myasthenic weakness of all limbs, such that the patient was dependent on a wheelchair and unable to raise his arms.

Laboratory diagnostic tests revealed elevated C-reactive protein (27.72 mg/dL [reference <0.50 mg/dL]) and gamma-glutamyl-transferase (491 U/L [reference <60 U/L]), with left shift of leukocytes and proteinuria. Other laboratory parameters were within reference limits. In cerebrospinal fluid, cell counts and protein concentration were within reference limits, but intrathecal IgG production was evident.

Real-time PCR on EDTA blood targeting the gene for the major outer membrane protein lip L32 of human pathogenic *Leptospira* spp. showed positive results. We then subjected the sample strain to multilocus sequence typing (MLST) (*4*) to investigate its relationship with other *L. interrogans* strains and determine whether the infection was acquired during the patient’s stay in Southeast Asia. In MLST analysis, we sequenced and analyzed 7 housekeeping genes of *Leptospira* spp. (*glmU*, *pntA*, *sucA*, *tpiA*, *pfkB*, *mreA*, and *caiB*). Among these, we found 2 new alleles (for *mreA* and *tpiA*), which resulted in a new sequence type (ST), 247. We constructed a phylogenetic tree for all 147 currently available *L. interrogans* STs, including ST247 (Figure 1) (*5*). The analysis showed that the isolate we obtained clusters with strains from Asia; however, the MLST database comprises strains predominantly from Asia. Further MLST analysis using eBURST (http://eburst.mlst.net) showed that linking STs were still missing. Further research in this field is required before a convincing phylogeographic conclusion can be reached.

**Figure 1:**
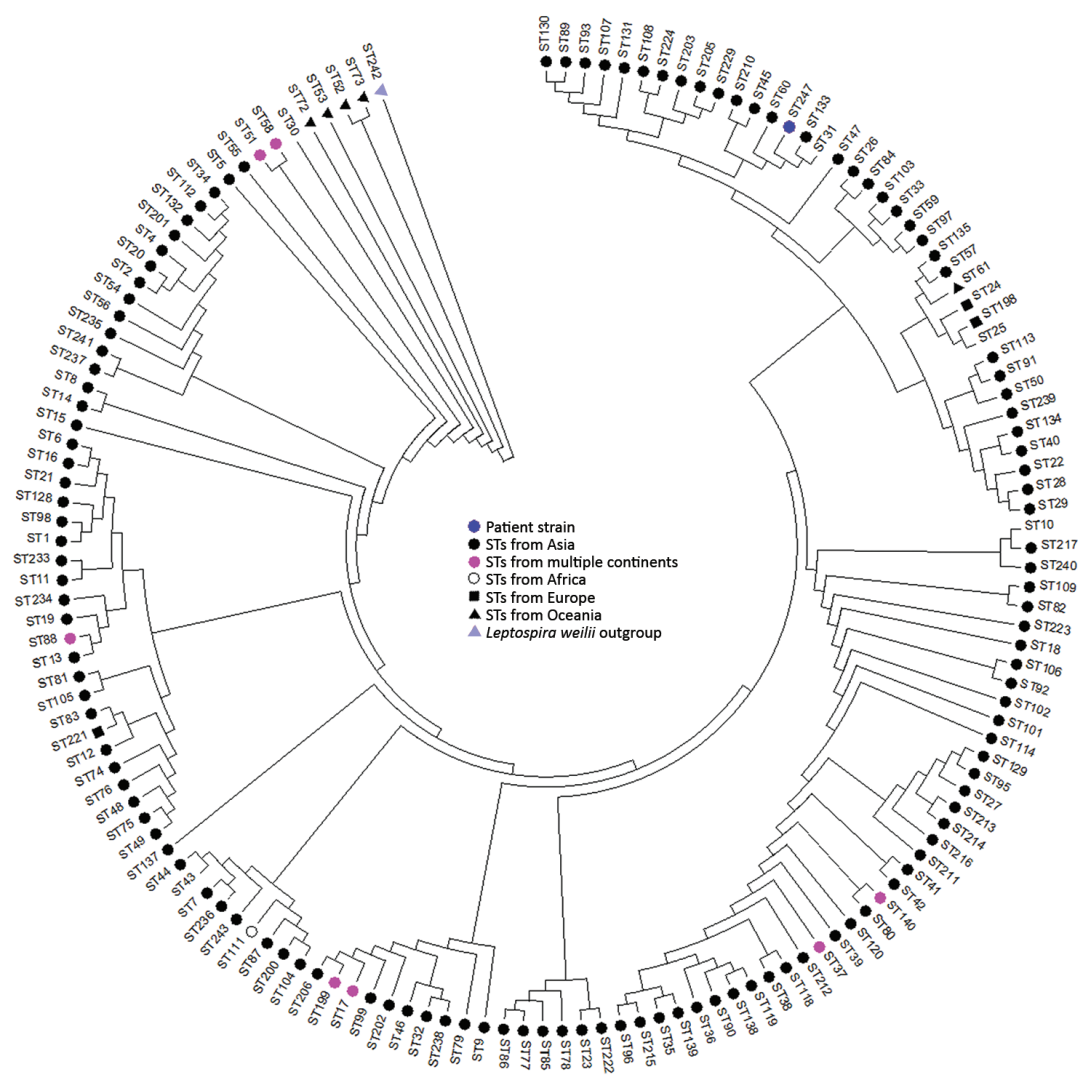
Phylogenetic tree showing only the topology for all *Leptospira interrogans* STs and their continent of origin. The isolate obtained from the patient in this study with severe myasthenic manifestation of leptospirosis is indicated by a blue dot (ST247). Tree was constructed using the maximum-likelihood method with MEGA version 7.0 (*5*). ST, sequence type.

No leptospiral DNA was found in the patient’s cerebrospinal fluid; no *Leptospira* spp. IgG or IgM were detected by ELISA (Virion/Serion, https://www.serion-diagnostics.de). Several days later, a second serum sample showed high levels of IgM (>100 U/mL).

The neurologic examination suggested MG, which was corroborated by pathologic decrements in compound muscle action potentials in the orbicularis oculi  (−15%) and trapezius (−46%) muscles on repetitive nerve stimulation at 3 Hz. Administration of the acetylcholinesterase inhibitor edrophonium resulted in strong and immediate improvement of ptosis, bulbar symptoms, and limb weakness. Nerve conduction studies revealed prolonged latency in the left medianus nerve, compatible with carpal tunnel syndrome. All other results were unremarkable, largely ruling out peripheral nerve or muscular disorders.

We consequently made a diagnosis of MG and acute leptospirosis and initiated treatment with pyridostigmine bromide and intravenous ceftriaxone. We withheld immunosuppressive therapy because of the acute leptospirosis. After 3 days, the patient demonstrated increased strength without further dysphagia or dysarthria. After 7 days, C-reactive protein levels normalized, proteinuria resolved, and gamma-glutamyltransferase levels decreased to 373 U/L. Ceftriaxone was discontinued after 10 days. Repetitive nerve stimulation was repeated 1 week after the initial test without prior ingestion of pyridostigmine and showed improved function of the neuromuscular junction.

We did not detect antibodies against acetylcholine receptors (AChRs) and muscle-specific tyrosine kinase (MuSK). However, testing for antibodies against low-density lipoprotein receptor-related protein 4 (LRP4) gave weakly positive results in a cell-based indirect immunofluorescence assay in an independent external laboratory (Medizinisches Versorgungszentrum Labor Volkmann Karlsruhe, Karlsruhe, Germany).

Pyridostigmine was discontinued, and the patient was discharged symptom-free and in good health. At follow-up 1 month later, examination of the patient found only minor ocular weakness. Repetitive nerve stimulation of the orbicularis oculi and trapezius muscles yielded normal results. At 4- and 6-month follow-up visits, minor fatigability of the lateral rectus muscle and the upper extremities was observed, but these effects did not impair the patient’s performance in a physically demanding profession and did not require medication. The timeline of the patient’s medical history is summarized in Figure 2.

**Figure 2:**
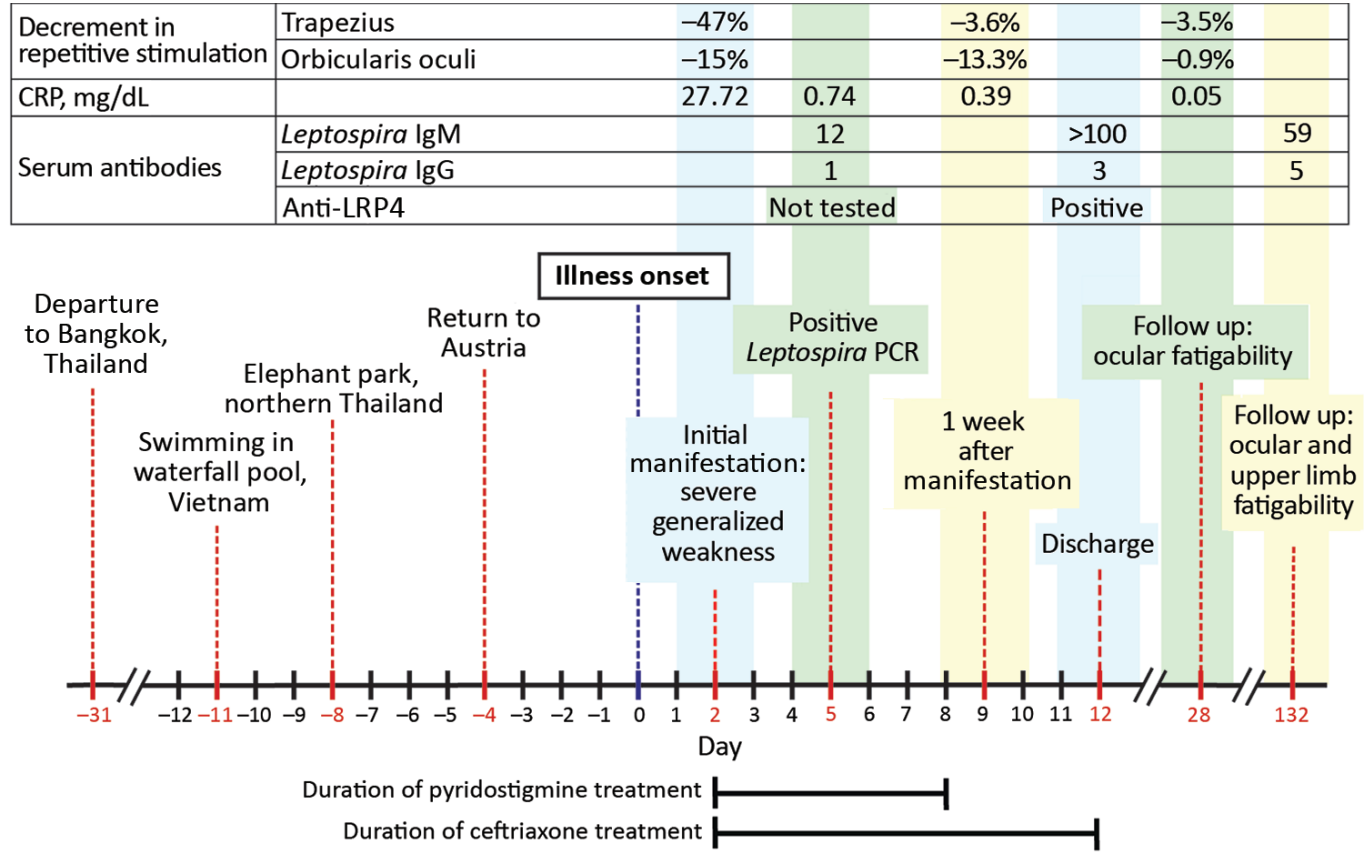
Timeline of medical history for patient with severe myasthenic manifestation of leptospirosis, including results of relevant neurologic and laboratory investigations. Decrement in repetitive stimulation denotes the maximum decrease in amplitude of the fourth or fifth compound muscle action potential waveform during supramaximal repetitive nerve stimulation at 3 Hz. A decrement >10% is regarded as pathologic (*6*). *Leptospira* ELISA cutoff values: IgG, <10 U/mL negative, 15 U/mL positive; IgM, <15 U/mL negative, >20 U/mL positive. CRP, C-reactive protein; LRP4, lipoprotein receptor-related protein 4.

MG is an acquired, antibody-mediated autoimmune disease of the neuromuscular junction causing muscle weakness and increased fatigability. Treatment consists of symptomatic relief with acetylcholinesterase inhibitors. Immunosuppression is often required for disease control. Approximately 85% of MG patients have AChR and MuSK antibodies (*7*); among the remaining patients, anti-LRP4 autoantibodies can be detected in 1.4%–50% of patients, depending on the detection method (*8*,*9*). Cell-based assays with live cells appear associated with lower frequencies compared with fixed-cell assays or with ELISA. Samples from the patient we describe tested negative for AChR and MuSK antibodies, but testing against LRP4 antibodies gave positive results in an external laboratory and borderline positive results in an in-house live cell-based assay (*10*). 

Our results should be interpreted cautiously. We were unable to reproduce them with remaining serum samples in the in-house live cell-based assay or ELISA at a later time.

Case series suggest an association of viral infections with the development of MG, although a causal link has yet to be shown (*11*,*12*). Worsening of preexisting MG is often triggered by an infection. A registry study in Spain found life-threatening events related to MG in up to 10% of patients, with infection being the most common cause (*13*).

The patient we report had a myasthenic crisis associated with a new ST of *L. interrogans.* Only 1 other case of leptospirosis with involvement of the neuromuscular junction has been described (*14*), but without direct identification of the pathogen and with symptoms occurring after the leptospiremic phase in the presence of *Leptospira*-specific antibodies; no myasthenic antibodies were tested for, and no diagnosis of MG was made.

In contrast, the patient we describe experienced more severe weakness at a much earlier point, and his serum sample tested positive for LRP4 autoantibodies, suggesting an association between MG and the acute leptospirosis. Reports of leptospirosis-associated, immune-mediated manifestations ranging from mononeuritis and Guillain-Barré–like syndromes to postinfectious autoimmune epilepsy (*3*,*15*) raise the question of whether *Leptospira* infections might precipitate or aggravate autoimmunity.
